# IL-36 Cytokines: Their Roles in Asthma and Potential as a Therapeutic

**DOI:** 10.3389/fimmu.2022.921275

**Published:** 2022-07-12

**Authors:** Hongna Dong, Yuqiu Hao, Wei Li, Wei Yang, Peng Gao

**Affiliations:** ^1^ Department of Respiratory Medicine, The Second Hospital of Jilin University, Changchun, China; ^2^ Department of Immunology, College of Basic Medical Sciences, Jilin University, Changchun, China

**Keywords:** IL-36, asthma, phenotype, inflammation, therapeutic agent

## Abstract

Interleukin (IL)-36 cytokines are members of the IL-1 superfamily, which consists of three agonists (IL-36α, IL-36β and IL-36γ) and an IL-36 receptor antagonist (IL-36Ra). IL-36 cytokines are crucial for immune and inflammatory responses. Abnormal levels of IL-36 cytokine expression are involved in the pathogenesis of inflammation, autoimmunity, allergy and cancer. The present study provides a summary of recent reports on IL-36 cytokines that participate in the pathogenesis of inflammatory diseases, and the potential mechanisms underlying their roles in asthma. Abnormal levels of IL-36 cytokines are associated with the pathogenesis of different types of asthma through the regulation of the functions of different types of cells. Considering the important role of IL-36 cytokines in asthma, these may become a potential therapeutic target for asthma treatment. However, existing evidence is insufficient to fully elucidate the specific mechanism underlying the action of IL-36 cytokines during the pathological process of asthma. The possible mechanisms and functions of IL-36 cytokines in different types of asthma require further studies.

## 1 Introduction

IL-36 cytokines are members of the IL-1 protein family, because the amino acid sequences of IL-36 cytokines have high similarity with IL-1β and IL-1Ra (1). The IL-36 cytokine family consists of three agonists (IL-36α, IL-36β and IL-36γ; previously named as IL-1F6, IL-1F8 and IL-1F9, respectively) and an antagonist, IL-36Ra (also termed as IL-1F5) (2). These agonists can bind to IL-36R (also called IL-1Rrp2) and IL-1RAcp, and recruit the myeloid differentiation primary response gene 88 (MyD88) signaling molecule to produce pro-inflammatory cytokines through the activation of nuclear factor−κB (NF−κB) and mitogen−activated protein kinase (MAPK) pathways (3). However, the engagement of IL-36R through antagonist IL-36Ra does not cause this effect (4). IL-36 cytokines are initially secreted as an inactive prerequisite form, truncating its N-terminus by proteolysis to become biological molecules (4). Studies have revealed that IL-36 cytokines can be cleaved by proteases. IL-36α is cleaved by elastase and cathepsin G, and IL-36β is cleaved by cathepsin G and protease-3 (5). IL-36γ can be cleaved by elastase, protease-3, cathepsin G and cathepsin S (5, 6). Notably, recent studies have revealed that microbially derived proteases can hydrolyze IL-36γ, which is critical for pathogen infection and inflammatory response (7, 8). The neutrophil-derived elastase can also cleave IL-36Ra (9). The biological activity and mechanism of IL-36 cytokine cleavage through proteases in specific disease microenvironments still need to be explored. This may help provide new directions for the elucidation of disease mechanisms and its treatment.

The IL-36 cytokine family is crucial for immune homeostasis and inflammation response through the regulation of the production of pro-inflammatory and anti-inflammatory cytokines (10–12). IL-36 cytokines can be secreted by different types of cells, including keratinocytes (13), macrophages (14), epithelial cells (15), T cells (16, 17), myofibroblasts (18), neutrophils (19), and plasma cells (20). Importantly, IL-36 cytokines contribute to the pathogenesis of autoimmune and inflammatory diseases, such as psoriasis, pulmonary diseases, inflammatory bowel disease, rheumatoid arthritis, allergic rhinitis, Sjogren’s syndrome, and systemic lupus erythematosus (15–17, 20–22). Recent studies have revealed that IL-36R is mainly expressed in lung fibroblasts, epithelial cells, dendritic cells, keratinocytes, eosinophils, macrophages, T cells, neutrophils, and B cells (10, 15, 23–28).

Asthma is a heterogeneous airway inflammatory disease characterized by airway inflammation and airway hyperresponsiveness (AHR) (29, 30). It is well-known that different subsets of T helper (Th) cells are critical for the pathogenesis of asthma (31, 32). In addition to classic Th1 and Th2 cells, a number of studies have suggested that Th9, Th17, Th22, and regulatory T cells (Tregs) are pivotal in asthma (33, 34). According to the ratio of eosinophils and neutrophils in induced sputum, asthma can be divided into four subtypes: paucigranulocytic asthma (PA), eosinophilic asthma (EA), mixed granulocytic asthma (MA), and neutrophil asthma (NA) (35). The treatment options for each subtype of asthma differ, and these subtypes of asthma varyingly respond to traditional therapies (35). Traditional EA is closely correlated to allergen-specific Th2 cells and their secreted cytokines (36), and well-responds to inhaled corticosteroids (37). In contrast, NA usually has a feature of neutrophil inflammation mediated by Th1 and Th17 cells, and is resistant to steroid therapies (38, 39), but responds to macrolide drugs (40, 41). Macrolide drugs have side effects. Hence, there is an urgent need to develop NA-targeted drugs. Therefore, it is important to identify the inflammatory subtypes of asthma in clinic, and novel biomarkers for its diagnosis and personalized treatment.

IL-36 cytokines play an important role in T cell response, and IL-36β can promote Th1 cell response in mice (10). In addition, IL-36β can cooperate with IL-12 to enhance the Th1 polarization *in vitro* (42). Furthermore, the engagement of IL-36R through its ligands can enhance the Th1 and Th17 response in *Aspergillus fumigatus*-infected peripheral blood mononuclear cells (PBMCs) (43). IL-36β promotes the development of M2 macrophages to produce IL-1β, tumor necrosis factor-α (TNF-α), IL-8, and other pro-inflammatory factors *in vitro* (25). Furthermore, IL-36 cytokines are potent regulators for neutrophilic and eosinophilic inflammation. Moreover, IL-36 cytokines can promote the production of IL-6 and CXC motif chemokine ligand (CXCL) 8 in human lung fibroblasts and bronchial epithelial cells, and enhance the inflammatory response of neutrophils (23). Interestingly, IL-36γ promotes eosinophil adhesion, migration and activation *in vitro*, and may be involved in the pathogenesis of allergic rhinitis *in vivo* (15). A complex relationship exists among IL-36 cytokines, nod-like receptor family pyrin domain 3 (NLRP3), neutrophil extracellular traps (NETs) and autophagy. *In vivo* and *in vitro* experiments have demonstrated that IL-36α promotes the NLRP3 expression and activation in mouse renal tubular epithelial cells and macrophages (44), while IL-36Ra inhibits NLRP3 activation and reduces inflammation in a mouse model of atherosclerosis (45). NETs increase the expression of IL-36α and IL-36γ in human bronchial epithelial cells (46). *In vitro* experiments have revealed that IL-36β activates autophagy in CD4^+^CD25^+^ Tregs, which may be valuable for the prognosis of sepsis (47). Accordingly, IL-36 cytokines play an important role in inflammation. However, further exploration is needed to determine whether these have the same role in asthma, or whether there are other unknown pathways. The present study summarizes the biological research advances in the roles of IL-36 cytokines in the pathogenic process of inflammatory subtypes of asthma, and uncovers new therapeutic targets for its personalized treatment.

## 2 IL-36 Cytokines and Inflammation

**Figure 1:**
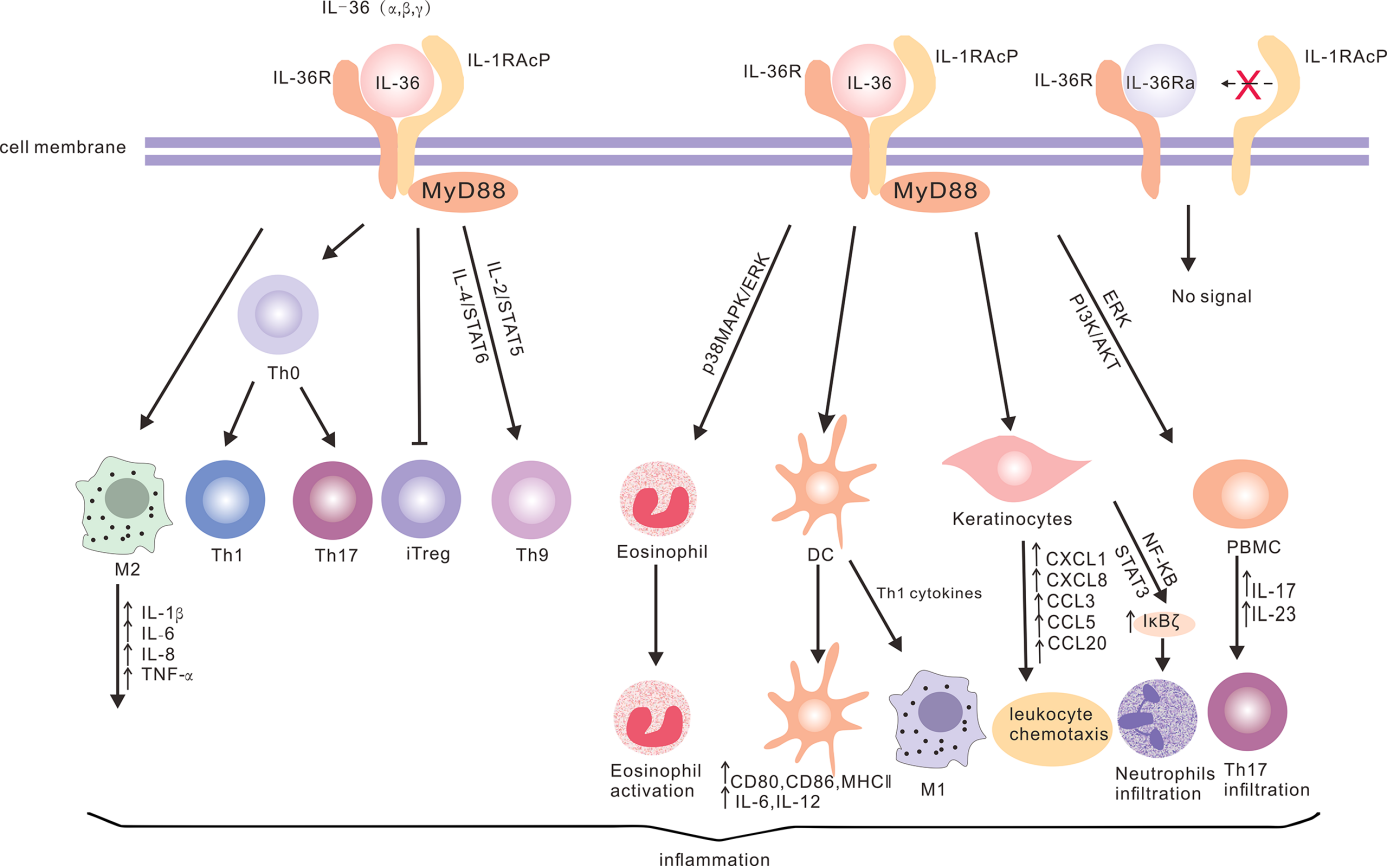
The production of IL-36 cytokines and the downstream signaling network. IL-36 can bind to IL-36R on a variety of cells (such as keratinocytes, dendritic cells, T cells, eosinophils, PBMCs and macrophages) through different signaling pathways to produce cytokines and chemokines, and promote neutrophil and eosinophil infiltration, T cell proliferation and macrophage polarization, in order to promote inflammation. However, IL-36Ra acts as an antagonist of IL-36R without activating the downstream signaling.

IL-36 cytokines are implicated in the pathogenesis of a number of diseases, such as allergic rhinitis (AR), chronic rhinosinusitis (CRS), inflammatory bowel disease, psoriasis, and rheumatoid arthritis (15, 20, 27, 48, 49). However, the expression levels and roles of IL-36 differ in these varying types of diseases, indicating the functional complexity in innate immunity and adaptive immunity (**Figure 1**). IL-36 cytokines may be involved in the pathogenesis of AR, since abnormal levels of serum IL-36 cytokines were detected in human AR patients (15). The levels of serum IL-36γ in AR patients are significantly higher, when compared to that in the control group, and this is positively correlated with the eosinophil count and eosinophilic cationic protein concentration (15). Indeed, IL-36γ can regulate the survival, migration, adhesion and activation of eosinophils *in vitro* (15). Similarly, higher levels of serum IL-36 cytokines were detected in AR patients, when compared to the control group, and IL-36α can promote Th17 cell differentiation *in vitro* (50). IL-36γ is highly expressed in CRS, and induces the production of chemokines to promote neutrophil inflammation *in vitro* (27). Furthermore, higher IL-36α expression levels were observed in patients with CRS and nasal polyps in the refractory group, when compared to the control group, and the increase in IL-36α expression and neutrophil inflammation were considered as risk factors for refractory in human CRS patients (51). Similarly, the IL-36α expression is higher in the rectal mucosa of patients with ulcerative colitis, when compared to that in healthy subjects, and IL-36 cytokines can regulate T cell subsets in a mouse model of intestinal inflammation (48). The treatment with recombinant IL-36 promotes Th1 responses, but inhibits Th17 responses *in vitro* (48). The IL-36 cytokine expression increased in a mouse model of psoriasis, and the reciprocal regulation between IL-36 cytokines and Th17-related cytokines maintained and amplified the pro-inflammatory response, contributing to the pathogenesis of psoriasis (49). In addition, IL-36α can enhance the expression of IL-6 and IL-8 in synovial fibroblasts *in vitro* (20).

IL-36 cytokines also contribute to the process of other airway inflammatory diseases. In chronic obstructive airway inflammation, these include chronic obstructive pulmonary disease (COPD) and asthma (52, 53). The induced sputum IL-36 cytokine levels in patients with eosinophilic airway inflammation were lower, when compared to patients with neutrophilic airway inflammation (53). Recent studies have revealed that the expression levels of IL-36Ra in asthma patients were lower, when compared to healthy controls, and that IL-36Ra can inhibit asthmatic inflammation in mouse models (54). Intrabronchial instillation with IL-36α induces an increase in CXCL1 and CXCL2, which recruit neutrophil infiltration in mice (55). Long-term smoking patients, with or without COPD, express higher levels of serum IL-36α, when compared to healthy non-smokers, and the serum IL-36α levels for these smoking patients are positively correlated with the concentrations of various pro-inflammatory cytokines (56).

IL-36 cytokines are an important hub, and have dual functions in pulmonary infectious diseases. These can not only act as immune defenders against infectious pathogens, but also contribute to lung injury. Lung IL-36γ levels increase in mice with bacterial pneumonia, and IL-36γ induces M1 macrophage activation and enhances the phagocytic activity, promoting bacterial clearance (57). Furthermore, the IL-36α and IL-36γ expression in the lungs can be induced by infection with *Pseudomonas aeruginosa*, and upregulate prostaglandin E2 production to impair bacterial clearance and deteriorate lung injury *in vitro* and *in vivo* (58). Moreover, the IL-36γ expression is elevated in *Mycobacterium tuberculosis*-infected macrophages, and enhances the macrophage’s phagocytosis of *Mycobacterium tuberculosis* (59). In addition, IL-36γ induces antimicrobial peptide production to limit the *Mycobacterium tuberculosis* replication in macrophages (60). Conversely, the IL-36 signaling appears to have a limited role in the host defense against *Mycobacterium tuberculosis* infection *in vivo* (61). Similarly, the IL-36α expression can be enhanced by influenza virus infection, and aggravate lung damage (62). In the mouse model of influenza virus-induced pneumonia, IL-36R deficiency mitigates mortality and lung damage, which is correlated to the reduction in the levels of pro-inflammatory factors, chemokines, and neutrophil aggregation (62). In addition, high IL-36γ expression levels promote the apoptosis of influenza A virus-infected lung epithelial cells, inhibit autophagy, and interfere with the IFN signaling pathway *in vivo* (63). IL-36γ is an important homeostatic regulator in antiviral response and viral immune evasion (63). The IL-36γ expression in airway epithelial cells can be enhanced by rhinovirus infection in asthmatic patients (64). Thus, the IL-36/IL-36R axis is correlated to the pathophysiology of airway inflammation. Further exploration is needed to elucidate the role of IL-36 cytokines in lung infection.

## 3 IL-36 Cytokines and Autophagy

Autophagy is a process of self-degradation of cells. This maintains cellular and organismal homeostasis by degrading its own organelles and proteins, and is critical for innate and adaptive immunity, and inflammatory response (65, 66). Furthermore, autophagy is involved in asthmatic inflammation, airway remodeling, and response to asthma treatment drugs (67–69). Autophagy-related 5 (*ATG5*) gene transcript variants are associated with the development of childhood asthma (70), and the polymorphisms of *ATG5* and *ATG7* are associated with neutrophilic airway inflammation in asthma (71). The increase in expression of *orosomucoid-like protein 3* (ORMDL3), a susceptibility gene, can induce autophagy in bronchial epithelial cells of asthmatic patients, and damage epithelial cells (72). Autophagy regulates innate and adaptive immunity during the development of asthma. Furthermore, autophagy regulates the survival and proliferation of innate lymphoid type-2 cells (ILC2s), and inhibiting the ILC2 autophagy can modulate ILC2 metabolic pathways, thereby inhibiting AHR and inflammation in allergic asthma (73). In asthmatic mice, IL-4 promotes B cell autophagy, and enhances the antigen presentation to CD4+ T cells, in order to exacerbate asthma (74). In recent years, a number of drugs with therapeutic potential for targeting autophagy (inhibiting autophagy or activating autophagy) in asthma have emerged. *In vitro*, budesonide and statins inhibit mTOR inhibitor-mediated autophagy in macrophages, decrease the Beclin-1 and LC3 expression, increase the p62 and IL-10 expression, and reduce asthmatic inflammation (69). Some Chinese herbal medicines can also regulate autophagy, and may be potential drugs for asthma treatment (75, 76). IL-36 cytokines are regulators of various inflammatory and immune diseases. It is noteworthy that there are few studies on the relationship between IL-36 cytokines and autophagy. A recent study revealed that IL-36 cytokines can improve rheumatoid arthritis symptoms by promoting synovial cell autophagy, thereby inhibiting synovial cell migration and invasion (77). In addition, IL-36β can enhance the autophagy of Tregs, and attenuate the immunosuppressive effect, improving the prognosis of patients with sepsis (47). Furthermore, IL-36γ can induce autophagy in cells infected with *Mycobacterium tuberculosis*, and enhance the bacterial clearance (59). Interestingly, IL-36γ promotes lung epithelial cell apoptosis and attenuates autophagy following influenza A virus infection, and enhances the antiviral immune response (63). Accumulating evidence has implicated autophagy in the pathogenesis of airway inflammation and airway remodeling in asthma (67, 78, 79). Accordingly, targeting autophagy through IL-36 cytokines may be important for the process of asthma, contributing to the complex pathogenesis of asthma. Further targeted therapy may be worthy of further exploration.

## 4 IL-36 in Human Asthma

**Figure 2:**
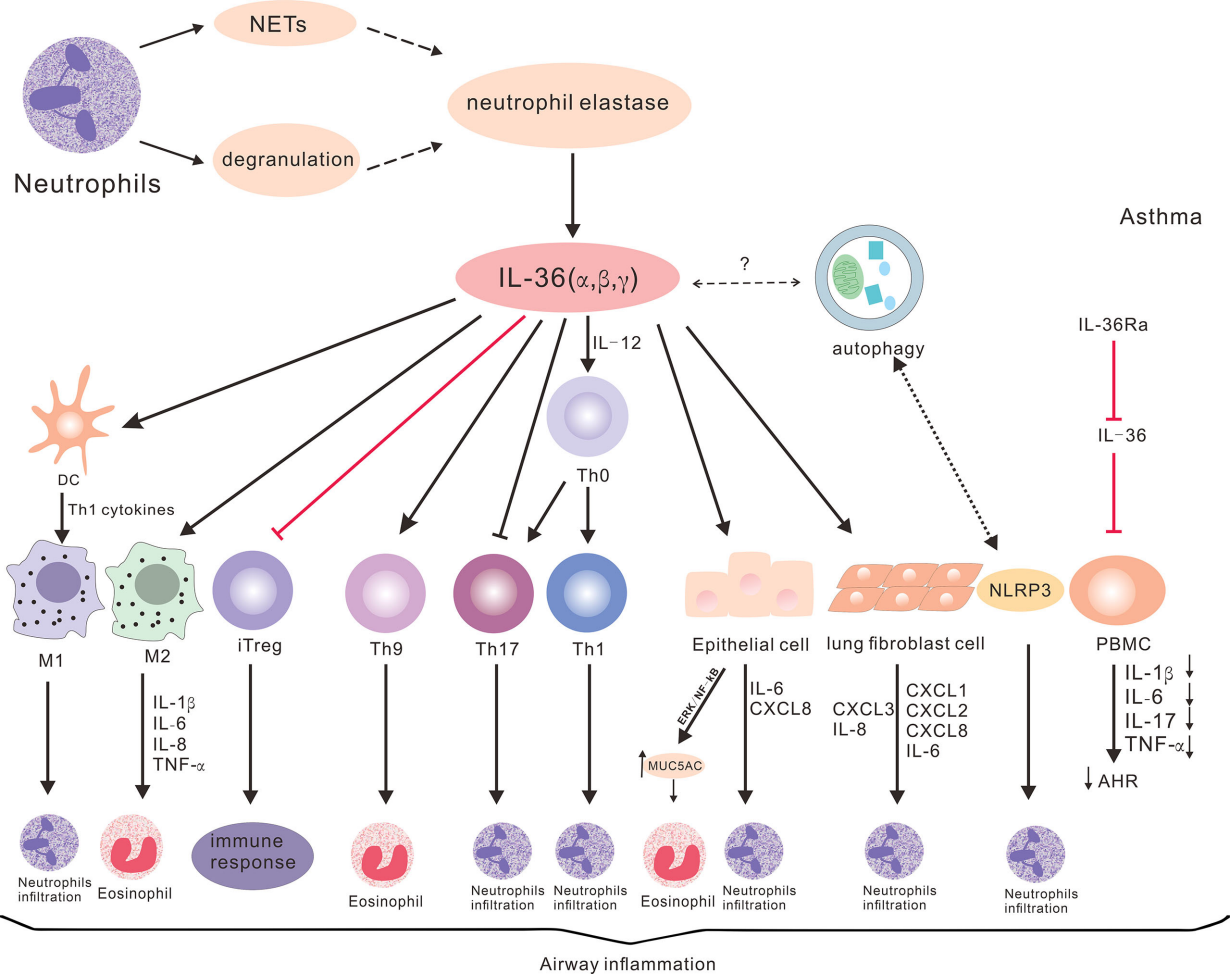
The potential roles of IL-36 cytokines in asthma. IL-36 cytokines promote Th1, Th17 and Th9 differentiation, and inhibit iTreg differentiation. These also enhance the production of cytokines and chemokines through lung epithelial cells, fibroblasts and macrophages, and promote neutrophil and eosinophil inflammation to participate in the pathogenesis of various subtypes of asthma. Neutrophil degranulation or NET-derived proteases can promote IL-36 cytokine cleavage, thereby promoting inflammatory response. IL-36 cytokines can also regulate autophagy and NLRP3, but the specific roles in asthma remain to be elucidated. IL-36Ra can reduce the production of cytokines, AHR and airway inflammation, and the inflammatory response of asthma.

The abnormal expression of IL-36 cytokines is involved in the pathogenesis of different subtypes of asthma (**Figure 2**). The mechanisms underlying the action of IL-36 cytokines in the pathogenesis of asthma may be complex through multiple channels. The IL-36 subfamily participates in the pathogenesis of different subtypes of asthma by secreting cytokines and chemokines for the recruitment and infiltration of T cells, neutrophils and eosinophils.

### 4.1 Abnormal Expression of IL-36 Cytokines in Asthma

Compared with healthy controls, the IL-36 expression is not significantly altered in patients with COPD and asthma (53). In contrast, when patients were further classified by inflammatory phenotype, COPD and asthma patients with eosinophilic airway inflammation have significantly lower levels of IL-36 cytokine expression, relative to healthy controls and patients with neutrophilic airway inflammation (53). The serum level of IL-36 cytokines is negatively correlated with the degree of eosinophilic inflammation, but is positively correlated with the degree of neutrophil inflammation in COPD and asthma patients (53). Similarly, the IL-36γ expression level is positively associated with the count of neutrophils in AR patients complicated with asthma (15). Conversely, the expression of IL-36Ra decreases, and IL-36Ra has anti-inflammatory effects in asthmatic patients, reducing the production of IL-1, IL-6, IL-17 and TNF-α in PBMCs and sputum cells (54). Furthermore, IL-36Ra reduces airway inflammation in asthmatic patients by inhibiting the activation of IL-36 signaling, and the production of pro-inflammatory factors (54).

### 4.2 The Mechanisms Underlying the Roles of IL-36 Cytokines in Asthma

The balance of different subsets of T cells is critical for regulating immune responses during the process of asthma. Th2 cells mediate eosinophilic asthma, and responds well to steroid therapy and novel biological agents (anti-IL-5, anti-IL-13, etc.), providing novel therapeutic strategies for this type of asthma (80, 81). Th17 cell-related factors promote smooth muscle cell contraction and proliferation for asthmatic airway remodeling and AHR (82, 83). Th1 and Th17 cells mediate neutrophilic asthma, and are associated with the resistance to steroid therapy (31, 84). Th9 cells and their secreting IL-9 increase in the peripheral blood and lung tissues of ovalbumin (OVA)-induced asthmatic mice (85). Th9 cells promote bronchial hyperresponsiveness in asthmatic mice, and are associated with the resistance to steroid therapy in asthmatic subjects (86, 87). Tregs have potent immunosuppressive activity. The frequency of circulating Treg cells in asthmatic patients was lower, when compared to that in healthy controls, and this was negatively correlated with asthma severity (88). The number of Tregs in the bronchoalveolar lavage fluid (BALF) of moderate-to-severe asthmatic patients was higher, when compared to that in healthy controls, which may reflect compensative anti-inflammatory response (89). However, another study revealed that the number of Tregs in BALF decreased in children with asthma (90). The treatment with iTregs can reduce airway inflammation and AHR in OVA-challenged asthmatic mice (91, 92). Th1/Th2 and Th17/Treg imbalance occur during the process of asthma (88, 93). The Th17/Treg ratio is positively correlated with the severity of AHR in children with asthma, and is associated with worsening asthma (93, 94). Huai Qi Huang (a mixture of Chinese herbs), ligustrazine and oligo-fucoidan can regulate the Th1/Th2 and Treg/Th17 imbalance in asthma, and reduce asthma severity (95–97). Given the essential role of T cells in asthma, researches that target the imbalance of different subsets of T cells show great potential. IL-36β and IL-12 promote Th1 polarization (42). The engagement of IL-36R through inflammatory mediators promote the Th1 response, and inhibit Th17 differentiation, regulation for intestinal inflammation and homeostasis in mice (48). IL-36α promotes the activation of the nucleotide-binding domain, the leucine-rich-containing family, and NLRP3 inflammasomes, enhances T cell proliferation and Th17 differentiation, and promotes tubular interstitial inflammation (44). Furthermore, the activation of IL-36R signaling can enhance the production of IL-17 and interferon-γ (IFN-γ) through Th17 and Th1 cells, respectively, in human PBMCs, after infection with *Aspergillus fumigatus* (43). IL-17A can feedback enhance the IL-36 expression, creating a positive cascade to increase the production of pro-inflammatory cytokines in human keratinocytes (49). The positive feedback effect of IL-17A and IL-36 also occurs in patients with CRS and nasal polyps (27). Th17 responses participate in the pathogenesis of NA (31, 98, 99). Th1 cells and their cytokines, as well as TNF-α, are the main mediators of NA, and are closely correlated to the resistance to steroid therapy (100, 101). Similar findings were achieved in the mouse model of neutrophil airway inflammation. Collectively, IL-36 cytokines promote Th1 differentiation, and have both promoting and inhibiting effects on Th17 differentiation, which are dependent on the specific inflammatory microenvironment in different types of inflammatory diseases.

In intestinal inflammation, IL-36γ can promote Th9 differentiation, which is mediated through the IL-2-STAT5 and IL-4-STAT6 pathways (102). IL-36γ inhibits inducible Treg differentiation by activating the NF-kB signaling (102). Th9 cells can induce eosinophil infiltration and bronchial hyperresponsiveness, independent of the eosinophils in mice (86), and this may be correlated to steroid resistance (87). In addition, some factors can enhance the Treg response to reduce the airway inflammation of asthma (103, 104). Apparently, the imbalance of Th9 and Treg response is crucial for the development of asthma, and the restoration of the balance between these may be applied as a novel therapeutic strategy for treating asthma. Indeed, epimedin C can regulate the Th9/Treg balance to inhibit airway inflammation in a mouse model of asthma (105).

In recent decades, the functions of IL-36 cytokines have been widely investigated, and abnormal IL-36 expression levels were associated with the development of a variety of diseases. Accordingly, IL-36 cytokines may be applied as therapeutic targets for the treatment of inflammatory diseases, including some types of asthma. Further understanding the molecular mechanisms underlying the actions of IL-36 cytokines in the inflammatory subtypes of asthma would provide a novel base for the design of personalized treatments for asthma. The potential mechanisms by which IL-36 cytokines contribute to the development of the inflammatory subtypes of asthma were further summarized.

### 4.3 IL-36 Cytokines Promote Inflammation in NA

#### 4.3.1 IL-36 Cytokines and NETs

Neutrophils are crucial for immune and inflammatory response. NET is the process of programmed neutrophil death, and pathogens can trigger NETs that comprise of extracellular DNA and multiple protein components, such as neutrophil elastase (NE) and myeloperoxidase, which are important for the clearance of bacterial pathogens (106). Interestingly, NETs are involved in the development of asthma, and are potential therapeutic targets. Furthermore, NETs are highly detected in NA mice, and promote neutrophilic inflammation (107). In NA mice, NETs can stimulate the production of CXCL1, CXCL2 and CXCL8 in lung airway epithelial cells, and promote the chemotactic infiltration of neutrophils (107). Furthermore, NETs stimulate macrophages to secrete IL-1β, recruit neutrophils, and further promote the progression of NETs (108). Moreover, NETs can stimulate airway epithelial cells to secrete pro-inflammatory factors, such as IL-1α, IL-1β and other IL-1 family members, thereby promoting the production of IL-36 cytokines, and further inducing neutrophil aggregation (46). In addition, NET-related proteases, cathepsin G and elastase can cleave IL-36 cytokines (109). Notably, the positive feedback loop between neutrophils and NETs promote the formation of NETs that act on keratinocytes through TLR4/IL-36R signaling to activate the MyD88/NF-kB signaling. This promotes the LCN2, IL-36γ, CXCL1 and CXCL8 expression, further recruiting neutrophils to deteriorate the inflammatory response (110).

#### 4.3.2 IL-36 Cytokines and Neutrophils

A recent report has revealed that IL-36 cytokines are important factors upstream of neutrophils (21). This is consistent with previous observations that IL-36 cytokines can recruit neutrophils by inducing CXCL1 and CXCL2 expression (55, 111, 112). Indeed, the levels of sputum IL-36α and IL-36γ in COPD patients are positively correlated with the number of sputum neutrophils (52). Consistently, higher levels of sputum IL-36 cytokines were observed in patients with neutrophil airway inflammation, and this was correlated with the number of sputum neutrophils, when compared to those with eosinophilic airways (53). IL-36α and IL-36γ increase the CXCL1 and CXCL2 expression to promote neutrophil recruitment *in vivo* (55, 111), and IL-36γ can activate the NF-kB signaling in macrophages, indicating that IL-36γ and IL-36α act as pro-inflammatory factors to recruit neutrophils (55, 111). Functionally, the injection of IL-36γ can increase the severity of AHR in mice (111). In generalized pustular psoriasis, IL-36 cytokines can promote keratinocytes to produce chemokines that recruit neutrophils (113). In addition, IL-36γ can promote the expression of neutrophil chemotactic factors of IL-8 and CXCL3 in lung fibroblasts (114).

#### 4.3.3 IL-36 Cytokines and NLRP3

NLRP3 is activated in HDM-induced asthmatic mice, and NLRP3 promotes Th2 response (115, 116). Dexamethasone can inhibit NLRP3 activation and attenuate the OVA-induced allergic airway inflammation in mice (117). The inhibition of NLRP3 is a promising strategy for the treatment of asthmatic inflammation (115, 118). IL-36 cytokines promote NLRP3 activation and IL-23/IL-17 production, leading to kidney inflammation and fibrosis (44). Another study revealed that IL-36Ra protects from atherosclerosis by inhibiting NLRP3 activation, and IL-1β and caspase-1 p10 production (45). The level of NLRP3 activation increased in NA patients (119), and this was associated with the degree of neutrophil airway inflammation, disease severity, and steroid resistance in asthma patients (120). In addition, there is a reciprocal regulatory relationship between autophagy and NLRP3. However, the precise mechanism underlying the action of NLRP3 in the pathogenesis of inflammatory asthma remains to be determined (121). In summary, IL-36 cytokines promote the pathogenesis of NA through multiple mechanisms.

#### 4.3.4 IL-36 Cytokines and Eosinophil Airway Inflammation

The level of sputum IL-36 cytokines in patients with eosinophilic obstructive airway inflammation was lower, when compared to that of neutrophil inflammation and healthy controls, and this was negatively correlated with the degree of eosinophil inflammation (53). Elevated IL-36β, IL-36γ and IL-36Ra expression levels can be detected in eosinophilic pustular folliculitis (122). IL-36γ promotes eosinophil inflammation through the activation of p38MAPK and MEK signaling *in vitro* (15). Recent data have indicated that the expression level of IL-36γ increases in AR patients, particularly in the asthmatic group, which is consistent with the findings for dermatophagoides pteronyssinus group 1, and that IL-17, IL-25 and IL-33 can upregulate the expression of IL-36R in eosinophils *in vitro* (15). IL-36 cytokines may trigger the aggregation of neutrophils and eosinophils, leading to tissue injury during the pathogenic process of autoimmune bullous dermatosis, such as dermatitis herpetiformis, bullous pemphigoid, and pemphigus vulgaris (123). Furthermore, IL-36 cytokines can regulate the pathogenesis of eosinophilic pustular folliculitis (122). Preliminary studies have revealed that IL-36 cytokines can inhibit eosinophilic airway inflammation, but promote eosinophilic inflammation in the process of other diseases. The effect of IL-36 cytokines on eosinophilic inflammation may depend on the pathogenic nature of the disease. The complex mechanisms underlying the roles of IL-36 cytokines remain to be elucidated.

#### 4.3.5 Regulation of IL-36 Cytokines on Monocytes/Macrophages

Macrophages can be classically activated as macrophages (M1) with pro-inflammatory activity, and alternately activated as macrophages (M2) with anti-inflammatory activities (124). Macrophage polarization is involved in asthma pathogenesis. M1 cells can secrete Th1-related cytokines and promote neutrophil inflammation, while M2 cells can promote Th2-dominant eosinophilic inflammation (125). Furthermore, macrophages can regulate the production of inflammatory factors involved in allergic asthma. In a mouse model of OVA-induced acute exacerbations, alveolar macrophages can promote the production of IL-1β, IL-6, CXCL-1 and TNF-α, exacerbating the asthmatic process (126). The increase in clearance of apoptotic cells through alveolar macrophages can help reduce the asthmatic inflammation (127), while the impaired macrophage function weakens the control of asthmatic inflammation (128). In addition, alveolar macrophages may be associated with the resistance to steroids in patients with severe asthma, when compared to non-severe asthma (129). IL-36 cytokines can modulate the functions of macrophages, contributing to the development of asthmatic airway inflammation. IL-36β enhances M2 cells to produce pro-inflammatory factors, such as IL-1β, IL-6, IL-8 and TNF-α, *in vitro* (25). Another study revealed that IL-36γ protects the body against tissue damage. In bacterial pneumonia, IL-36γ promotes the production of Th1-related cytokines by DCs, which enhance the classical activation of macrophages into M1 cells (57). In renal tubule interstitial lesions, IL-36 cytokines promote the activation of NLRP3 inflammasome in macrophages, contributing to the pathogenesis of renal disease (44). In a mouse model of dextran sodium sulfate (DSS)-induced colitis, IL-36α can promote colonic inflammation by enhancing neutrophil and macrophage infiltration (48). The regulation of macrophage polarization by IL-36 cytokines in specific environments is complex, and exerts different effects, which needs to be further studied.

#### 4.3.6 The Effect of IL-36 Cytokines on Other Types of Cells

IL-36 cytokines can promote the production of IL-6 and CXCL8 in lung fibroblasts and bronchial epithelial cells *in vitro* (23). CXCL8 is an inducer of neutrophils, and promotes NA and inflammation. IL-6 can promote NA resistance to corticosteroids (38). Through extracellular signal regulated kinase (ERK) 1 and 2, p38, and NF-kB signaling in human airway epithelial cells, IL-36γ increases the expression of MUC5AC, and plays a role in allergic and inflammatory diseases (130). The MUC5AC expression was upregulated in mouse models of EA and NA (131). MUC5AC is crucial for airway eosinophil inflammation and AHR, and the levels of sputum MUC5AC in patients with mild asthma before steroid treatment were higher, when compared to that in the healthy control group (132). IL-36β can promote monocyte-derived DC maturation and induce Th1 inflammatory response through IL-1Rrp2 (133). IL-36γ can be expressed through lung fibroblasts, and activate the downstream signaling to produce neutrophil chemokines IL-8 and CXCL3, and Th17 chemokine CCL20 (114). TNF-α, IL-1β, IL-17, and the TLR3 agonist dsRNA can stimulate IL-36γ production in airway epithelial cells *in vitro*, which exacerbates neutrophil inflammation (114).

## 5 IL-36 Cytokines in Animal Models of Asthma

IL-36 cytokines have a pro-inflammatory activity in mouse models of asthma. In asthmatic A/J mice induced by ovalbumin, the IL-36γ expression levels were upregulated (134). Recent data has revealed that treatment with IL-36Ra to target IL-36R reduces airway inflammation by inhibiting the activation of NF-kB signaling in the mouse model of OVA-induced asthma (54). Similarly, higher levels of IL-36γ, CXCL1 and CXCL2 were detected in the lung of asthmatic mice, following the challenge with house dust mite extracts (111). In addition, intratracheal infusion with IL-36γ can increase the severity of AHR in mice (111). Similarly, the allergen challenge increases the CXCL1 mRNA transcripts, and neutrophil infiltrates in the BALF in the mouse model of OVA-induced asthma (135). Furthermore, IL-36γ can enhance the NF-kB expression in mouse lungs (111), and sustain the NF-kB activation in patients with severe uncontrolled asthma, promoting the production of inflammatory mediators during the pathogenic process of asthma (136).

## 6 Asthma Treatment and IL-36 Cytokines

IL-36 cytokines have a central role in autoimmune diseases. Serum IL-36α and IL-36γ levels were elevated in patients with systemic lupus erythematosus, when compared to the healthy groups, and these were positively correlated with complement C3 levels (22). Furthermore, the IL-36γ expression was upregulated in psoriatic tissues and serum (137). IL-36 cytokines promote the production of TNF-α, IL-6 and IL-8 pro-inflammatory factors in keratinocytes, and IL-36 and Th17 are present in the feedback loop to promote psoriatic inflammation (49). IL-36α induces psoriatic dermatitis in mice, and the administration of the anti-IL-36R monoclonal antibody can significantly attenuate the psoriatic skin thickening and inflammation (138). In addition, IL-36α was elevated in the intestinal mucosa of patients with ulcerative colitis, when compared to healthy controls, further demonstrating the reduced inflammation in IL-36R deficient DSS-induced colitis mice (48). In mice with intestinal fibrosis, the mucosal inflammation was reduced, and the fibrosis attenuated the anti-IL-36R antibody (139). IL-36 cytokines play an important role in the pathogenesis of autoimmune diseases, and blocking the IL-36/IL-36R axis is a promising therapeutic strategy for the treatment of autoimmune diseases.

The IL-36/IL-36R signaling may serve as a potential target for future treatments, including neutralizing antibodies against IL-36 cytokines and the IL-36 receptor, and inhibitors of enzymes for IL-36 cytokine cleavage. IL-36 inhibitors can directly act on IL-36 cytokines. The IL-36γ inhibitor (A-552) can bind human IL-36γ to inhibit its pro-inflammatory effects, which was validated in a human psoriasis model of IL-36γ-induced 3D skin equivalents (140). In addition, since IL-36 cytokines require cleavage to exert their active effects, the inhibition of proteases that cleave IL-36 cytokines is another promising strategy. A study revealed that a small-molecule inhibitor of elastase can inhibit IL-36γ cleavage and subsequent inflammatory responses (141). Another study reported that peptide-based cathepsin G or elastase inhibitors that target IL-36 cytokines can inhibit their cleavages, and exert anti-inflammatory effects in psoriasis (142).

Targeting IL-36R is also an effective approach to block IL-36R related signaling. The anti-IL-36R antibodies of ANB019 (imsidolimab) and BI655130 (spesolimab) are presently included in clinical trials. Spesolimab is a selective humanized antibody against the IL-36 receptor that inhibits the activation and response of downstream pro-inflammatory signaling (143). In a single-arm, phase I study, the spesolimab blockade of IL-36R exhibited the rapid regulation of deregulated molecular pathways in generalized pustular psoriasis and palmoplantar pustulosis, IL-36, Th1/Th17, and neutrophil-mediated and keratinocyte-mediated inflammation-related genes were downregulated at the first week after spesolimab administration (144). A recent study reported a single, open-label phase I trial for the intravenous administration of 10 mg/kg of spesolimab for seven patients with generalized pustular psoriasis (145). The disease severity was assessed using GPPGA scores at the start of the trial, and five patients achieved clear or nearly clear skin within the first week (145). The skin of all patients, with or without IL-36Ra mutation, achieved clear or almost clear skin at the fourth week (145). Spesolimab has been proven to be effective, although mild-to-moderate adverse effects can occur (145).

Another 12-week, phase IIa, multicenter, double-blind, randomized controlled clinical trial was conducted to test the therapeutic effects and safety of spesolimab in 59 patients with palmoplantar pustulosis, following the treatment with 900 mg or 300 mg of spesolimab or placebo (146). The severity of palmoplantar pustulosis decreased at a faster rate over time in the spesolimab treated group, when compared to the placebo group (146). These results indicate that spesolimab has a potential therapeutic effect, and can be well-tolerated with comparable adverse events (146). ANB019 has been tested in phase I clinical trials on healthy volunteers (147), and is being evaluated in a clinical trial for generalized pustular psoriasis patients (148). IL-1RAcP (IL-1R3) is an attractive therapeutic target. Antibodies against IL-1R3 have been evaluated in multiple preclinical models, and blocking IL-1R3 inhibits several factors of the IL-1 family, including IL-36, thereby reducing inflammation in the disease (149). More research is needed to explore the significance of this target in the future.

Peptide-based neutrophil protease inhibitors and small-molecule elastase inhibitors have the characteristics of low cost and can be applied locally in the diseased skin (141, 142). However, the efficient and specific synthesis of protease inhibitors remains challenging (150). The application value of its systemic administration for disease treatment is worth exploring in future research. Currently, biological drugs targeting cytokines such as IL-4, IL-5, IL-13, and TNF-α have been approved or are under development for the treatment of uncontrolled severe asthma (151, 152). The strategies for neutralization of a cytokine are very effective, but they are expensive, and given the cost-effectiveness, more severe uncontrolled asthma is more suitable for such inhibitors (141, 152). In addition, biological drugs require systemic administration, which may cause adverse effects, such as infection and allergic reactions, and the optimal delivery method of such drugs to the target site remains to be studied (152). A study has shown that individuals with the IL-36R gene knockout mutations does not impair the immune defense function, suggesting that blocking the IL-36 signaling may have good safety (153). Targeting the IL-36 cytokine pathway is promising for the treatment of neutrophilic asthma or more severe asthma in the absence of other appropriate therapies, although more researches are needed in the future.

Furthermore, the modulation of NETs and autophagy may also be promising strategies to inhibit airway inflammation that involve IL-36 cytokines. NETs and autophagy modulators are potential therapeutic reagents for asthma. Peripheral blood neutrophil autophagy and NETs commonly occur in severe asthmatic patients, and can damage airway epithelial cells to promote inflammation (154). The inhibition of the NET process may help reduce asthmatic inflammation, and nitric oxide synthase can inhibit the formation of NETs (155). The therapeutic peptide P140 to downregulate the autophagic flux can attenuate AHR and airway inflammation in allergic mice (156, 157), and inhibit NET release (157). Therefore, further investigation on the network of IL-36 cytokines in the pathogenesis of asthma is warranted, which may provide a potentially promising strategy for asthma treatment.

## 7 Discussion

IL-36 cytokines can inhibit eosinophilic airway inflammation, promote neutrophil inflammation, and participate in the pathogenesis of different subtypes of asthma. These may be applied as novel therapeutic targets for the treatment of asthma. Through the receptors, IL-36 cytokines can regulate different types of inflammatory cells, including neutrophils, eosinophils, macrophages and DCs. The specific mechanism underlying the actions of IL-36 cytokines in different subtypes of asthmatic inflammation remains not fully understood. Hence, further research is needed.

In conclusion, IL-36 cytokines have important biological roles in the development and progression of asthma. IL-36 cytokines regulate the differentiation of T lymphocytes, and functions of neutrophils, eosinophils and macrophages, induce the production of cytokines and chemokines, leading to the infiltration of neutrophils and eosinophils, and participate in the pathogenesis of various subtypes of asthmatic inflammation. However, the exact mechanism underlying the actions of IL-36 cytokines in different subtypes of inflammatory asthma remains unclear. Accordingly, strategies that target the IL-36 cytokine family may help in the design of novel therapies for the treatment of asthma.

## Author Contributions

HD drafted and revised the manuscript. YH, WL and WY collected the information and conducted the literature review. PG conceived and designed the study. All authors contributed to the article and approved the submitted version.

## Funding

This review was supported by grants from the Natural Science Foundation of Jilin Province (20210101460JC) and the National Natural Science Foundation of China (82070037).

## Conflict of Interest

The authors declare that the research was conducted in the absence of any commercial or financial relationships that could be construed as a potential conflict of interest.

## Publisher’s Note

All claims expressed in this article are solely those of the authors and do not necessarily represent those of their affiliated organizations, or those of the publisher, the editors and the reviewers. Any product that may be evaluated in this article, or claim that may be made by its manufacturer, is not guaranteed or endorsed by the publisher.

## Abbreviations

AHR, airway hyperresponsiveness; AR, allergic rhinitis; ATG5, Autophagy-related 5 gene; BALF, bronchoalveolar lavage fluid; COPD, chronic obstructive pulmonary disease; CRS, chronic rhinosinusitis; CXCL, CXC motif chemokine ligand; DCs, dendritic cells; DSS, dextran sodium sulfate; EA, eosinophilic asthma; ERK, extracellular signal regulated kinase; HDM, house dust mite extract; IFN-γ, interferon-γ; IL, Interleukin; IL-36Ra, IL-36 receptor antagonist; ILC2s, innate lymphoid type-2 cells; iTreg, inducible regulatory T; MA, mixed granulocytic asthma; MAPK, mitogen−activated protein kinase; MyD88, Myeloid differentiation primary response gene 88; NA, neutrophilic asthma; NE, neutrophil elastase; NETs, neutrophil extracellular traps; NF−κB, nuclear factor−κB; NLRP3, nod-like receptor family pyrin domain containing 3; ORMDL3, orosomucoid-like protein 3; OVA, ovalbumin; PA, paucigranulocytic asthma; PBMCs, peripheral blood mononuclear cells; TNF-α, tumor necrosis factor-α; Th, T helper.
